# Macroglossia and less advanced dystrophic change in the tongue muscle of the Duchenne muscular dystrophy rat

**DOI:** 10.1186/s13395-022-00307-7

**Published:** 2022-10-19

**Authors:** Keitaro Yamanouchi, Yukie Tanaka, Masanari Ikeda, Shizuka Kato, Ryosuke Okino, Hiroki Nishi, Fumihiko Hakuno, Shin-Ichiro Takahashi, James Chambers, Takashi Matsuwaki, Kazuyuki Uchida

**Affiliations:** 1 Laboratory of Veterinary Physiology, Graduate School of Agricultural and Life Sciences, The University of Tokyo, 1-1-1 Yayoi, Bunkyo-ku, Tokyo, 113-8657 Japan; 2 Laboratory of Veterinary Pathology, Graduate School of Agricultural and Life Sciences, The University of Tokyo, 1-1-1 Yayoi, Bunkyo-ku, Tokyo, 113-8657 Japan; 3 Laboratory of Animal Cell Regulation, Graduate School of Agricultural and Life Sciences, The University of Tokyo, 1-1-1 Yayoi, Bunkyo-ku, Tokyo, 113-8657 Japan

**Keywords:** Duchenne muscular dystrophy, Masseter muscle, Tongue, Macroglossia, Rat

## Abstract

**Background:**

Duchenne muscular dystrophy (DMD) is an X-linked muscle disease caused by a complete lack of dystrophin, which stabilizes the plasma membrane of myofibers. The orofacial function is affected in an advanced stage of DMD and this often leads to an eating disorder such as dysphagia. Dysphagia is caused by multiple etiologies including decreased mastication and swallowing. Therefore, preventing the functional declines of mastication and swallowing in DMD is important to improve the patient’s quality of life. In the present study, using a rat model of DMD we generated previously, we performed analyses on the masseter and tongue muscles, both are required for proper eating function.

**Methods:**

Age-related changes of the masseter and tongue muscle of DMD rats were analyzed morphometrically, histologically, and immunohistochemically. Also, transcription of cellular senescent markers, and utrophin (*Utrn*), a functional analog of dystrophin, was examined.

**Results:**

The masseter muscle of DMD rats showed progressive dystrophic changes as observed in their hindlimb muscle, accompanied by increased transcription of *p16* and *p19*. On the other hand, the tongue of DMD rats showed macroglossia due to hypertrophy of myofibers with less dystrophic changes. Proliferative activity was preserved in the satellite cells from the tongue muscle but was perturbed severely in those from the masseter muscle. While *Utrn* transcription was increased in the masseter muscle of DMD rats compared to WT rats, probably due to a compensatory mechanism, its level in the tongue muscle was comparable between WT and DMD rats and was similar to that in the masseter muscle of DMD rats.

**Conclusions:**

Muscular dystrophy is less advanced in the tongue muscle compared to the masseter muscle in the DMD rat.

**Supplementary Information:**

The online version contains supplementary material available at 10.1186/s13395-022-00307-7.

## Background

Duchenne muscular dystrophy (DMD) is an X-linked muscle disease caused by out-of-frame mutations in the dystrophin gene that results in the complete loss of dystrophin protein [9]. Dystrophin is a structural protein that stabilizes the plasma membrane of the myofibers, and the lack of dystrophin protein causes myofibers to become fragile [9]. Skeletal muscle myofibers normally have a remarkable regenerative capacity [11]. Once myofibers are damaged, the myogenic stem/progenitor cells, satellite cells, are activated and proliferate, then eventually fuse each other to form multinucleated myotubes. The myotubes mature to constitute newly regenerated myofibers. DMD is characterized by repeated cycles of degeneration and regeneration of myofibers, which finally result in a loss of regenerative capacity due to exhaustion of the satellite cell pool [21]. DMD patients show progressive weakness and premature death due to a loss of ambulation [9].

In an advanced stage of DMD, an orofacial function is affected [3] and eating disorders such as dysphagia are often a problem [37]. Dysphagia is caused by multiple etiologies. Decreased mastication is one of the causes of dysphagia. Masticatory movement involves masseter, temporalis, medial, and lateral pterygoid muscles [17]. Masticated foods are then swallowed using the tongue muscle in the oral cavity. Swallowing is initiated by the contraction of digastric and tongue muscles against the palatine to move the bolus into the oropharynx, followed by a complex coordination of pharyngeal and esophageal muscles. The tongue muscles consist of the extrinsic muscles, which move the tongue from the outside, and the intrinsic muscles, which change the shape of the tongue. The former consists of four types of tongue muscles: the genioglossus, hyoglossus, styloglossus, and palatoglossus muscles, and the latter consists of four types: the superior longitudinal, inferior longitudinal, transverse, and vertical muscles. Several reports demonstrated that dysphagia in DMD patients is associated with dystrophic changes in muscles related to mastication [36]. Macroglossia, which is common in DMD patients [28, 37], is defined as an enlarged tongue and is often associated with dysphagia [5]. Therefore, preventing the deterioration of mastication and swallowing functions in DMD is important to improve the patient’s quality of life.

We previously reported the generation of a rat model of DMD (DMD rat) [22, 33]. DMD rats show a progressive muscle pathology in their hindlimb muscle and diaphragm like human DMD [22, 33]. The pathological changes seen in the muscle of DMD rats include decreased strength, increased fibrosis, and fatty infiltration [22, 33]. We further demonstrated the occurrence of premature cellular senescence in the muscle of DMD rats and suggested that this could be one of the causes of satellite cell depletion, which leads to a loss of regenerative capacity [33].

In the above our studies using DMD rats [22, 33], we analyzed only the muscle of the hindlimbs and diaphragm. During breeding and maintenance of DMD rats, we had experienced a decreased food intake in accordance with the decrease in the body weight in their later life, and this prompted us to examine whether the muscles related to food intake are also affected. Thus, in this study, we focused on the masseter and tongue muscles as the muscles involved in mastication and swallowing and investigated their development and histological changes, as well as the dynamics of satellite cells.

## Methods

### Animals

We previously generated and characterized a strain of DMD rat [22, 33]. In this strain of DMD rat, a deletion of 329 bp around the splice site in intron2 leads to exon3 skipping, and an insertion of 1 bp in exon16 causes the generation of a stop codon, resulting in a loss of dystrophin protein [22, 33]. Adult X^Dmd^X female rats were mated with wild-type (WT) male rats to generate male WT (XY) and DMD (X^Dmd^Y) rats. They were maintained under controlled environmental conditions, at 23 °C with a light/dark cycle (lights on 0800–2000). Laboratory chow (Labo MR Standard, Nihon Nousan Co., Yokohama, Japan) and water were given ad libitum. All animal experiments performed in this study were in accordance with the Guide for the Care and Use of Laboratory Animals of the University of Tokyo and were approved by the Institutional Animal Care and Use Committee of the University of Tokyo (P18-125).

### Measurement of food intake

At 3 or 7 months old, the rats were transferred individually to a plastic cage and their amount of food intake was measured for two consecutive days. The measured values were divided by 2 and expressed as food intake per day.

### Histological and immunohistochemical analyses

**Fig. 1:**
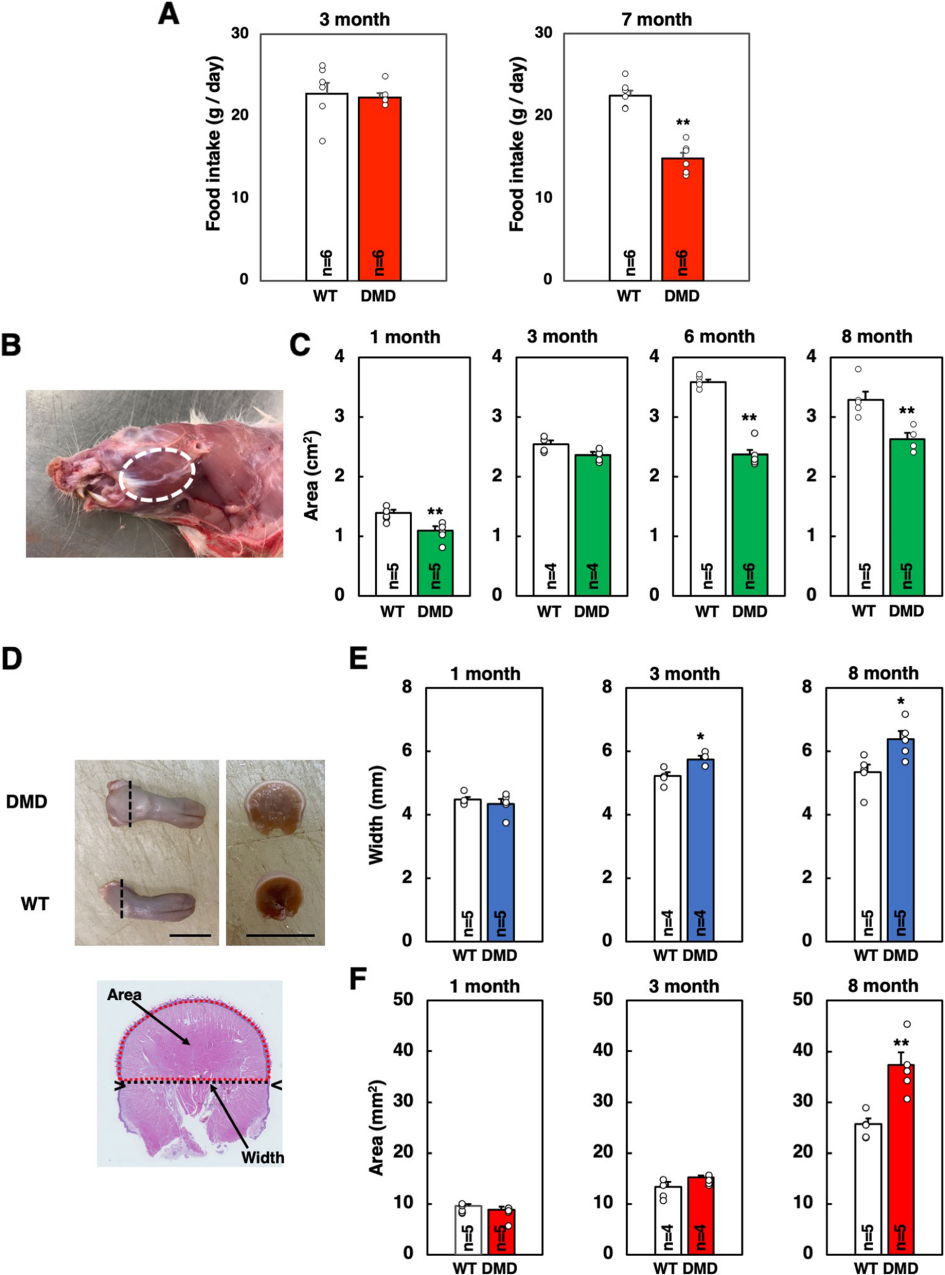
Food intake and morphometrical analyses of masseter and tongue muscles of WT and DMD rats. **A** The amount of food intake per day was measured at 3 and 7 months old. The data are expressed as mean ± SE. **, *p* < 0.01 by unpaired Student’s *t*-test. **B** The masseter muscle was considered as an ellipse (dotted white circle) and its long and short diameters were measured with calipers to calculate the area. **C** Changes in the area of the masseter muscle at 1, 3, 6, and 8 months old. The data are expressed as mean ± SE. **, *p* < 0.01 by unpaired Student’s *t*-test. **D** The tongue was cut at the root (dotted black line) (left) and the width (dotted black line) and area (dotted red circle) were measured. Scale bar = 1 cm. **E** Changes in the width and cross-sectional area of the tongue at 1, 3, and 8 months old. The data are expressed as mean ± SE. **, *p* < 0.01 by unpaired Student’s *t*-test

At indicated age, the rats were killed by inhalation of carbon dioxide gas. After removal of facial skin, the area corresponding to the masseter muscle was measured. The masseter muscle was considered as an ellipse (Fig. 1B), and its long and short diameters were measured with calipers to calculate the area. Then, the lower half part of the masseter muscle was removed. The tongue was cut out at the root. Collected tissues were fixed in 4% paraformaldehyde (PFA) in phosphate-buffered saline (PBS) for paraffin-embedded sections or snap-frozen in liquid nitrogen-cooled isopentane for cryosections.

The paraffin-embedded sections were used for hematoxylin–eosin (HE) or Masson’s trichrome staining. The sections were observed and photographed using a microscope (BX51, Olympus, Tokyo, Japan) equipped with a digital camera (DP73, Olympus). The HE-stained tongue sections were used for measurement of the width and area of the tongue (Fig. 1D). Masson’s trichrome-stained sections were used for quantitative analysis of the fibrotic area. Two fields were randomly selected in the section using a 4 × objective and the area occupied by fibrotic tissues stained blue and the total area of sections were calculated using ImageJ software (v1.47; National Institutes of Health, Bethesda, MD, USA).

The cryosections (7 μm) were used for immunohistochemistry. The sections were fixed with 4% PFA in PBS for 15 min, followed by blocking with 5% normal donkey serum (NDS) in PBS. The sections were incubated with primary antibodies overnight at 4 °C, then secondary antibodies for 1 h.

For identification of satellite cells, vascular endothelial cells, and mesenchymal progenitor cells, anti-Pax7 (1:200 with 5% NDS in PBS, mouse, clone P3U1; Developmental Studies Hybridoma Bank (DSHB), Iowa City, IA, USA), anti-CD31 (1:400 with 5% NDS in PBS, rabbit, NB100-2284, Novus Biological, Centennial, CO, USA), and anti-chondroitin sulfate proteoglycan 4 (CSPG4) (1:50 with 5% NDS in PBS, mouse, clone 5C12 [34]) were used as primary antibodies, respectively, followed by AlexaFluor-labeled donkey anti-mouse IgG (1:500 with 5% NDS in PBS, Jackson ImmunoResearch, West Grove, PA, USA).

For identification of necrotic myofibers, anti-laminin (1:400 with 5% NDS in PBS, rabbit, L9393 Sigma, St Louis, MO, USA) was used as the primary antibody, and AlexaFluor-labeled goat anti-rat IgG and donkey anti-rabbit IgG (1:500 with 2.5% NDS/2.5% normal goat serum (NGS) in PBS, Jackson ImmunoResearch, West Grove) were used as secondary antibodies.

For identification of newly formed regenerated myofibers, anti-embryonic myosin heavy chain (eMHC) (1:400 with 5% NDS in PBS, mouse, clone F1.652, DSHB) and anti-laminin were used as primary antibodies, and AlexaFluor-labeled donkey anti-mouse IgG and donkey anti-rabbit IgG (1:500 with 5% NDS in PBS, Jackson ImmunoResearch) were used as secondary antibodies. After the reaction with secondary antibodies, nuclei were counterstained with Hoechst 33258. The sections were observed and photographed using a microscope equipped with a digital camera.

For quantification of Pax7-positive cells, IgG-positive necrotic myofibers, and embryonic myosin heavy chain (eMHC)-positive myofibers, several numbers of the field were photographed using × 4 or × 10 objectives, and the number of positive cells or myofibers were counted. Myofibers were identified by double-staining with an anti-laminin antibody. Data were expressed as percent positive myofibers or number of positive cells per area.

For quantification of myofiber size and the number of nuclei per myofiber, the sections were immunostained with anti-laminin, followed by labeling with AlexaFluor-labeled secondary antibody. The nuclei were counterstained with Hoechst 33258. The sections were photographed as described above. The myofibers were automatically identified by Cellpose Google Colab script (https://colab.research.google.com/drive/1958UQIH-XAYogKvbxnaUHALYvR73KLj2) [38], and their diameters (minimum Feret diameters) and numbers were measured using ImageJ software. The number of nuclei per myofiber was calculated by dividing the total number of nuclei by the number of myofibers.

The area occupied by CD31-positive cells and CSPG4-positive cells was calculated by ImageJ.

### Cell culture and immunocytochemistry

Mononuclear cells were isolated from skeletal muscle as described previously [39]. In brief, the rats were killed by inhalation of carbon dioxide gas, and their tongue and masseter muscles were extirpated. As described above, the lower half part of the masseter muscle and tongue cut out at the root were used for cell isolation. They were minced with scissors and digested with 1.25 mg/mL protease (from *Streptomyces griseus*, type XIV; Sigma) at 37 °C for 1 h. Cells were separated from myofiber fragments through differential centrifugation and plated onto poly-l-lysine- and fibronectin-coated 48-well plates. Cells from each muscle piece were divided into two equal portions (one for immunocytochemistry of Pax7 and another for MyoD) and plated. Cells were cultured in Dulbecco’s modified Eagle medium (Gibco, Life Technologies, Palo Alto, CA, USA) containing 10% fetal bovine serum, 100 U/mL penicillin, 100 μg/mL streptomycin, and 50 μg/mL gentamicin for 4 days.

The cells were fixed with 4% PFA/PBS for 15 min and then blocked with 5% NGS/PBS containing 0.1% Triton X-100 for 10 min. The cells were incubated with anti-Pax7 (1:200 with 5% NGS/PBS) or anti-MyoD (1:200, with 5% NGS/PBS, mouse, clone 5.8A, Novocastra, Newcastle upon Tyne, UK) overnight at 4 °C. After washing, they were incubated with AlexaFluor-labeled secondary antibody for 1 h. Nuclei were counterstained with Hoechst 33,258. The Pax7- and MyoD-positive cells were counted in randomly selected five fields at 20 × objective of a fluorescence microscope (BX50, Olympus). The total numbers of nuclei were also counted. The data were expressed as percent positive cells.

### Quantitative real-time PCR

Total RNA was extracted from 100 cryosections (7 μm) with FastGene RNA Basic kit (Nippon Genetics Co., Ltd., Tokyo, Japan) and reverse transcribed to cDNA using Super Script II kit (Invitrogen). Quantitative real-time PCR was performed on a Light Cycler 2.0 (Roche Diagnostics, Roche, Basel, Switzerland) with the Thunderbird SYBR qPCR Mix (TOYOBO, Osaka, Japan). The following primer sets were used: *p16*: forward, 5′-TTC ACC AAA CGC CCC GAA CA-3′; reverse, 5′-CAG GAG AGC TGC CAC TTT GAC-3′; *p19*: forward, 5ʹ-GTG TTG AGG CCA GAG AGG AT-3ʹ; reverse, 5ʹ-TTG CCC ATC ATC ATC ACC T-3ʹ; *p21*: forward, 5ʹ-GAC ATC TCA GGG CCG AAA-3ʹ; reverse, 5ʹ-GGC GCT TGG AGT GAT AGA AA-3ʹ; *p53*: forward, 5ʹ-AGA GAG CAC TGC CCA CCA-3ʹ; reverse, 5ʹ-AAC ATC TCG AAG CGC TCA C-3ʹ; *Utrn*: forward, 5ʹ‐TAG AGC AAT ACG CCA CAC GA‐3ʹ; reverse, 5ʹ‐ACG CTC TTC CTT CTC CAC AG‐3ʹ; *MuRF1*: forward, 5ʹ‐AGG ACT CCT GCC GAG TGA C-3′; reverse, 5′-TTG TGG CTC AGT TCC TCC TT-3′; *Atrogin1*: forward, 5′-GAA GAC CGG CTA CTG TGG AA-3′; *Atrogin1*: reverse, 5′-ATC AAT CGC TTG CGG ATC T-3′, and *HPRT*: forward, 5ʹ‐GAC CGG TTC TGT CAT GTC G‐3ʹ; reverse, 5ʹ‐ACC TGG TTC ATC ATC ACT AAT CAC‐3ʹ. The expression of each gene was analyzed using the crossing‐point method and expressed after normalization with that of *HPRT*.

### Western blotting

Two hundred cryosections (7 μm) of the tongue muscle were homogenized using radioimmunoprecipitation assay (RIPA) buffer [10 mM NaH_2_PO_4_, 150 mM NaCl, 2 mM ethylenediaminetetraacetic acid (EDTA), 0.1% sodium deoxycholate, 1% Nonidet P-40, 10 μg/mL leupeptin, 5 μg/mL pepstatin, 1.84 g/L Na_3_VO_4_, 10 mg/mL *p*-nitrophenylphosphate (PNPP), 100 KIU/mL aprotinin, 20 μg/mL phenylmethanesulfonylfluoride (PMSF)], and protein concentration of the lysates was determined using Pierce BCA Protein Assay Kit (Thermo Fisher Scientific, Waltham, MA, USA). Lysates were, then, diluted with RIPA buffer to the same concentrations of protein. The samples were subjected to sodium dodecyl sulfate–polyacrylamide gel electrophoresis (SDS-PAGE) and western blotting as described in the previous study [1]. Antibodies used were as follows: anti-phospho-Akt antibody (Ser473, Cell Signaling Technology, Danvers, MA, USA, #9271), anti-Akt antibody (Cell Signaling Technology, #9272), anti-phospho-p70 S6K antibody (Thr389, Cell Signaling Technology, #9234), anti-p70 S6K antibody (Santa Cruz Biotechnology, C-18, sc-230), anti-phospho-S6 ribosomal protein antibody (Ser240/244, Cell Signaling Technology, #2215), anti-S6 ribosomal protein antibody (Cell Signaling Technology, 54D2, #2317), anti-4EBP1 antibody (Cell Signaling Technology, #9452), anti-α-tubulin antibody (Sigma Aldrich, T9026), anti-rabbit IgG, horseradish peroxidase (HRP)-linked whole antibody from donkey (GE healthcare, NA934V), and anti-mouse IgG, HRP-linked whole antibody from sheep (GE healthcare, NA931V). Band intensities of each blot were quantified using ImageJ.

### Statistical analyses

Except for an analysis of myofiber diameters, graphed data are expressed as means ± SE. Unpaired Student’s *t*-test (between two groups) and one-way analysis of variance (ANOVA) followed by a Tukey–Kramer test (for multiple group comparison) were used to evaluate statistical differences. For the experiment to assess the difference of myofiber diameters, the *p*-value was determined using the Wilcoxon rank sum test. *p*-values less than 0.05 were considered statistically significant.

## Results

We previously reported that the body weight of DMD rats decreased progressively after the age of 6 months [33]. Suspecting that eating disorder occurs in DMD rats, we measured the amount of food intake at the ages of 3 months (before the onset of body weight decrease) and 7 months (after the onset of bodyweight decrease). The amount of food intake at 3 months old was comparable between WT and DMD rats (Fig. 1A). However, at 7 months old, significantly decreased food intake was observed in DMD rats, indicating the occurrence of an eating disorder (Fig. 1A).

The above results suggest that muscles involved in food intake are affected by the progress of the disease. We then performed morphometric analyses of muscles involved in food intake. We chose the masseter and tongue muscles because these two muscles are to be approached easily and distinguishable from other muscles. As shown in Fig. 1B, the area corresponding to the masseter muscle was considered as an ellipse, and its long and short diameters were measured with calipers to calculate the area. The area of the masseter muscle was measured from 1 to 8 months old. At 1 month old and after 6 months old, the area of the masseter muscle was significantly reduced in DMD rats compared to WT rats. Especially, the greater reduction was observed at 6 and 8 months old (Fig. 1C).

The appearance of the tongue at 8 months old is shown in Fig. 1D. The cross-section of the posterior part of the tongue was larger in DMD rats than in WT rats (Fig. 1D). Then, we measured the area and width of the upper half of the tongue sections. Quantitative analysis showed that the width of the tongue of DMD rats was significantly larger than that of WT rats after 3 months old (Fig. 1E). In addition, the area of the tongue was significantly larger in DMD rats at the age of 8 months than in WT rats (Fig. 1F). These results indicate that macroglossia is occurring in DMD rats.

**Fig. 2:**
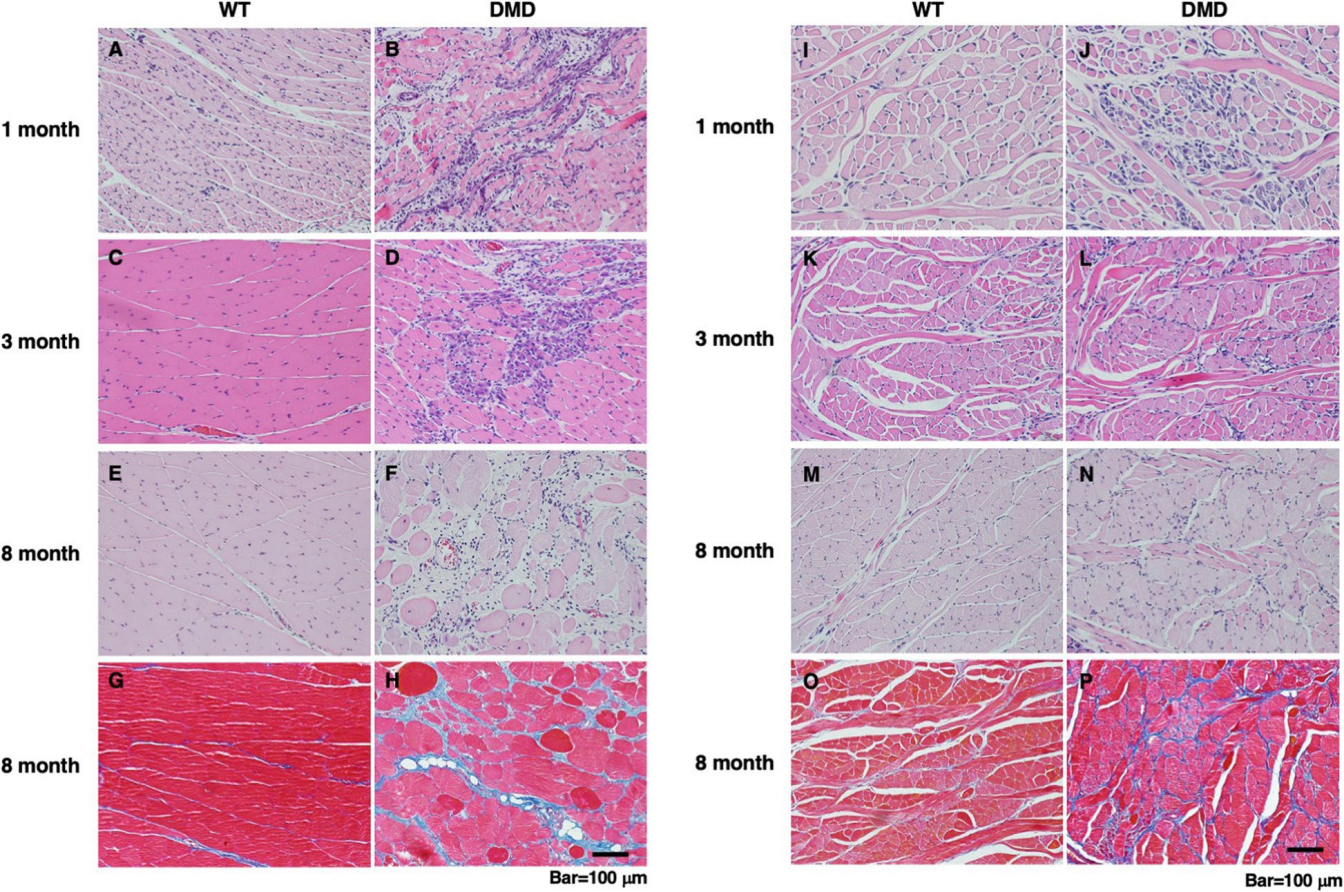
Histological analyses of masseter and tongue muscles of WT and DMD rats. **A**–**F** HE- and **G**,** H** Masson’s trichrome-stained sections of the masseter muscle of WT and DMD rats. In the masseter muscle of DMD rats, myofiber degeneration and necrosis with infiltration of neutrophils and macrophages at 1 month old, regenerative myofibers with enlarged centralized nuclei and basophilic cytoplasm at 3 months old, and severe fibrosis, confirmed by Masson’s trichrome staining, at 8 months old were observed, respectively. No significant pathological changes were observed in the masseter muscle of WT rats at all ages. **I**–**N** HE- and **O**, **P** Masson’s trichrome-stained sections of the tongue muscle of WT and DMD rats. In the tongue muscle of DMD rats, regenerative myofibers with enlarged centralized nuclei and basophilic cytoplasm at 1 month old, the regenerative changes accompanied by mild fibrosis at 3 months old, and persistence of mild fibrosis, confirmed by Masson’s trichrome staining, at 8 months old were observed, respectively. No significant pathological changes were observed in the tongue muscle of WT rats at all ages

To further gain insight into the age-related changes of the masseter and tongue muscles of DMD rats, histological analyses were performed. For the tongue muscles, the intrinsic and extrinsic muscles were examined separately. The masseter muscle of DMD rats showed myofiber degeneration and necrosis with infiltration of neutrophils and macrophages at 1 month old (Fig. 2B) and regenerative myofibers with enlarged centralized nuclei and basophilic cytoplasm at 3 months old (Fig. 2D). At 8 months old, in addition to inflammatory and degenerative changes, severe fibrosis and fatty infiltration were observed (Fig. 2F, H). No significant pathological changes were observed in the masseter muscle of WT rats at all ages (Fig. 2A, C, E, G).

In contrast, in the intrinsic tongue muscle of DMD rats, regenerative myofibers with enlarged centralized nuclei and basophilic cytoplasm were observed at 1 month old (Fig. 2J), and these regenerative changes were accompanied by mild fibrosis at 3 months old (Fig. 2L). At 8 months old, mild fibrosis, also confirmed by Masson’s trichrome stain (Fig. 2P), was persisted (Fig. 2N). The extrinsic tongue muscles generally showed similar findings as to the intrinsic tongue muscles, although regenerative changes were observed in some parts of the genioglossus muscles of DMD rats at 3 and 8 months old (Supplementary Fig. 1). No significant pathological changes were observed in both the intrinsic and extrinsic tongue muscle of WT rats at all ages (F ig. 2I, K, M, O).

**Fig. 3:**
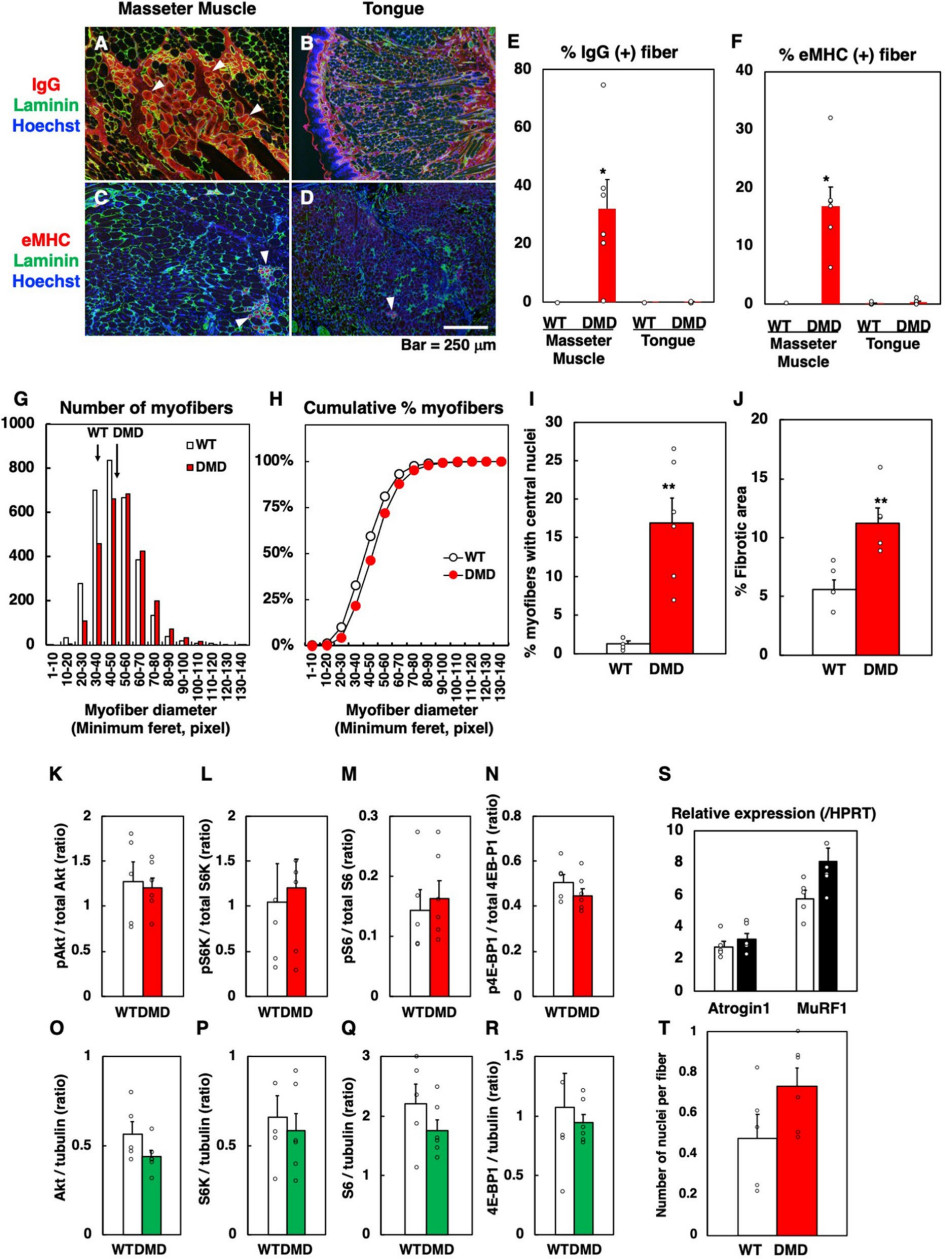
Analyses of necrotic and regenerating myofibers in the masseter and tongue muscle of WT and DMD rats (**A**–**F**). **A**, **B** Representative immunohistochemistry of endogenous IgG (red) and laminin (green) in the masseter (**A**) and tongue (**B**) muscle of DMD rats at 6 months old. Nuclei were stained with Hoechst 33258 (blue). Arrowheads (white) indicate necrotic myofibers. **C**, **D** Representative immunohistochemistry of embryonic myosin heavy chain (eMHC) (red) and laminin (green) in the masseter (**A**) and tongue (**B**) muscle of DMD rats at 6 months old. Nuclei were stained with Hoechst 33258 (blue). Arrowheads (white) indicate regenerating myofibers. **E** Quantitative analysis of necrotic and regenerating myofibers in masseter and tongue muscle of WT (*n*=5) and DMD (*n*=6) rats at 6 months old. The data are expressed as mean±SE. *, *p*<0.05 vs others by Tukey–Kramer test. Analyses of tongue muscle of WT and DMD rats (**G**–**J**). **G**, **H** Distribution of myofiber diameters at 6 months old. The left panel (**G**) depicts the relative distribution of myofibers with certain diameters. The right panel (**H**) depicts relative cumulative plots based on the data shown in the left panel. The myofiber diameters of DMD rat (*n*=6) were larger than those of WT rats (*n*=5). *p*<0.01 by Wilcoxon rank sum test (number of myofibers analyzed, WT=3112 and DMD=2662). **I** Ratio of myofibers with central nuclei at 6 months old. The data are expressed as mean±SE. **, *p*<0.01 by unpaired Student’s *t*-test. **J** Relative fibrotic area at 6 months old. The data are expressed as mean±SE. **, *p*<0.01 by unpaired Student’s *t*-test. Quantitative analyses of molecules involved downstream of Akt-mTOR pathway in the tongue muscle of WT (*n*=5) and DMD rats (*n*=6) at 6 months old (**K**–**R**). **K**–**N** Ratio of phosphorylated Akt, S6K (S6 kinase), S6 (S6 ribosomal protein), and 4E-BP1. **O**, **P** Total amount of Akt, S6K (S6 kinase), S6 (S6 ribosomal protein), and 4E-BP1 normalized to that of α-tubulin. The data are expressed as mean±SE. Quantitative analyses of expression of two muscle-specific E3 ubiquitin ligases (Atrogin1 and MuRF1) in the tongue muscle of WT (*n*=5) and DMD rats (*n*=6) at 6 months old (**S**). The data are expressed as mean±SE. Quantitative analysis in the number of nuclei per myofiber of the tongue muscle of WT (*n*=5) and DMD rats (*n*=6) at 6 months old (**T**). The data are expressed as mean±SE

To further examine whether myofiber necrosis and muscle regeneration are ongoing at 6 months old when macroglossia is occurring in the tongue muscle of DMD rats, immunohistochemical analyses of endogenous IgG (myofiber necrosis) and embryonic MHC (muscle regeneration) were performed. The masseter muscle of DMD rats, though there is variation among individuals, showed marked myofiber necrosis (Fig. 3A, E) and muscle regeneration (Fig. 3C, F), while the intrinsic tongue muscle showed none of these (Fig. 3B, D–F). These results indicate that, although the masseter and tongue muscles of DMD rats undergo pathological changes that are not seen in WT rats, the changes seen in the tongue muscle are much milder than in the masseter muscle of DMD rats.

To investigate the factors that are responsible for macroglossia seen in DMD rats, we measured the myofiber diameters of the intrinsic tongue muscle of WT and DMD rats at 6 months old. The myofiber diameters of DMD rats were larger than those of WT rats (Fig. 3G, H; *p* < 0.01 by the Wilcoxon rank sum test). The ratio of myofibers with central nuclei was significantly higher in DMD rats than in WT rats, indicating that muscle regeneration had occurred in the past (F ig. 3I). In addition, quantitative analysis of Masson’s trichrome-stained sections as shown in Fig. 2 reveals that the fibrotic area in the tongue muscle of DMD rats is about twice that of WT rats (Fig. 3J). Thus, these results suggest that both the hypertrophic response of myofibers and the accumulation of collagen between myofibers were combined to produce macroglossia. Considering the possibility that cells present in the stromal area are affected, the number of vascular endothelial cells and mesenchymal progenitor cells in the tongue muscle was compared between WT and DMD rats at 6 months old. The area occupied by CD31-positive (endothelial) and CSPG4-positive (mesenchymal progenitor) cells were comparable (Supplementary Fig. 2).

Hypertrophy of myofibers occurs by two mechanisms: increased protein synthesis through activation of the PI3K-Akt-mTOR pathway and the addition of new myonuclei by fusion of satellite cells [12]. To gain more insight into the mechanism of hypertrophy of myofibers observed in the tongue muscle of DMD rats, we investigated the amount and phosphorylation of molecules that act in the downstream of PI3K-Akt-mTOR pathway as well as the number of nuclei per myofiber in 6-month-old tongue muscle. No differences were observed between WT and DMD rats in the amount and phosphorylation of S6K, S6, and 4E-BP1 (Fig. 3K–R; Supplementary Fig. 3). Neither the expression nor the phosphorylation of Foxo1, which is thought to be involved in protein degradation, could be detected (data not shown). In addition, no significant differences were observed in the transcription of two skeletal muscle-specific ubiquitin ligase genes, *Atrogin 1* and *MuRF1*, which are involved in protein degradation [2] (Fig. 3S). When the number of nuclei per myofiber in transverse sections was calculated, a trend toward a higher number of nuclei was observed in the DMD rats, although this was not statistically significant (Fig. 3T). These results suggest that, at least at the age of 6 months, there is neither an increase in protein synthesis nor a suppression of protein degradation and that an increased number of fused satellite cells is more likely involved in the hypertrophy of myofibers.

**Fig. 4:**
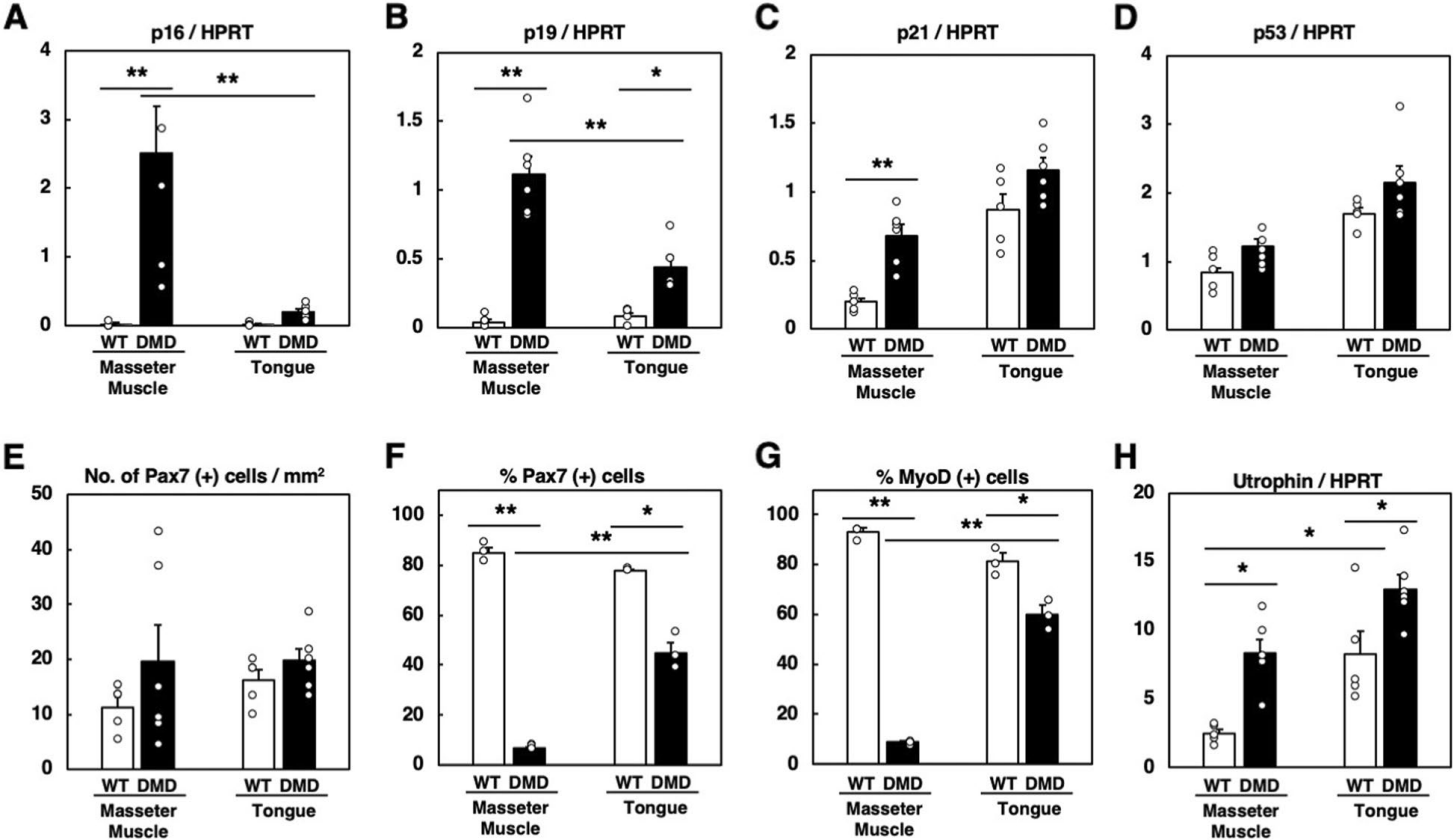
Quantitative analyses of gene expression and satellite cells in muscles of WT and DMD rats. **A** *p16*, **B** *p19*, **C** *p21*, and **D** *p53* transcriptions in the masseter and tongue muscle at 9 months old. The data are expressed as mean ± SE. * and **, *p* < 0.05 and *p* < 0.01 by unpaired Student’s *t*-test, respectively (WT, *n* = 5; DMD, *n* = 6). **E** Number of Pax7-positive satellite cells in the sections at 9 months old. The data are expressed as mean ± SE. There was no significant difference among all groups by Tukey–Kramer test (WT, *n* = 5; DMD, *n* = 6). **F**,** G** Relative number of Pax7- and MyoD-positive satellite cells from 9-month-old rats after 4-day culture in vitro. The data are expressed as mean ± SE. * and **, *p* < 0.05 and *p* < 0.01 by unpaired Student’s *t*-test, respectively (WT, *n* = 3; DMD, *n* = 3). **H** *Utrn* transcription in the masseter and tongue muscle at 9 months old. The data are expressed as mean ± SE. *, *p* < 0.05 by unpaired Student’s *t*-test (WT, *n* = 5; DMD, *n* = 6)

We reported previously that cellular senescence with increased transcription of *p16* and *p19* occurs in the hindlimb muscles of DMD rats and that the proliferation of muscle satellite cells is markedly suppressed in culture [33]. Therefore, we examined the transcription of senescence-associated genes in the masseter and tongue muscles at 9 months old. *p16* and *p19* were markedly upregulated in the masseter muscle of DMD rats compared to WT rats (Fig. 4A, B). On the other hand, both *p16* and *p19* were slightly upregulated in the tongue muscle of DMD rats but the degrees were significantly lower than those in the masseter muscle (Fig. 4A, B). *p21* transcription was significantly upregulated only in the masseter muscle of DMD rats (Fig. 4C), and there was no difference in the *p53* transcription in both the muscles between WT and DMD rats (Fig. 4D). There was no significant difference in the number of Pax7-positive satellite cells in the sections, although there was a large variation between individuals (Fig. 4E). On the other hand, when cultured in vitro for 4 days, the number of Pax7- or MyoD-positive satellite cells derived from the masseter muscle of DMD rats was significantly lower than that of WT rats (Fig. 4F, G, Supplementary Fig. 4A and B), while the number of satellite cells derived from the tongue muscle of DMD rats was only slightly lower than that of WT rats (Fig. 4F, G, Supplementary Fig. 4A and B). These results indicate that cellular senescence, possibly through p16- and p19-dependent pathway, occurs extensively in the masseter muscle of DMD rats as was seen in their hindlimb muscles [33], while it is significantly less severe in the tongue muscle.

The above results suggest that the dystrophic changes are less advanced in the tongue muscle of DMD rats. Utrophin, known as dystrophin-related protein, had been shown to compensate for the function of dystrophin [7, 13, 20, 26]. In a mouse model of DMD, mdx mouse, the skeletal muscle phenotype is milder than that seen in DMD patients [20], and this had been once thought to be due to an upregulation of utrophin [20]. Thus, we examined the *Utrn* transcription in the masseter and tongue muscles of WT and DMD rats at 9 months old. In the masseter muscle, *Utrn* transcription was significantly upregulated by the absence of dystrophin (Fig. 4H). Interestingly, in the tongue muscle, *Utrn* transcription was also upregulated but its basal expression in the presence of dystrophin (WT rats) was comparable to that in the masseter muscle of DMD rats (Fig. 4H). This result suggests that the tongue muscle is intrinsically expressing a higher level of *Utrn* transcription even in the presence of dystrophin, and this might be related to the resistance of this tissue against degenerative changes in the absence of dystrophin.

## Discussion

In the present study, we demonstrated that the tongue of DMD rats shows macroglossia and degenerative changes were observed transiently only in the early period of life. On the other hand, chronic degenerative and necrotic changes were seen in the masseter muscle of DMD rats.

Cellular senescence is caused by oxidative stress due to chronic inflammation [6]. We previously reported progressive myofiber degeneration and necrosis, along with cellular senescence with increased transcription of *p16* and *p19*, in the tibialis anterior (TA) and diaphragm of DMD rats [33]. *p16* transcript was mainly present in satellite cells and mesenchymal progenitor cells, and their proliferative activity was severely suppressed in vitro. The phenotype of the masseter muscle of DMD rats seen in this study is similar to our previous reports; the masseter muscle showed progressive myofiber degeneration and necrosis, and accordingly, *p16* and *p19* transcription was upregulated. In contrast, the transcription of *p16* and *p19* in the tongue muscle of DMD rats was much lower than those in the masseter muscle, and the proliferative activity of the satellite cells was relatively well-preserved in vitro. This is in agreement with the histological observation that myofiber necrosis and inflammation were not observed in the tongue muscle of DMD rats, suggesting the absence of oxidative stress that could induce cellular senescence.

A similar phenotype to the tongue muscle of DMD rats had been observed in the hindlimb muscle of mdx mice [10, 19, 35]. In the tongue muscle of DMD rats, regeneration of myofibers was transiently observed at 1 month old, and at later periods of their life, myofibers with central nuclei persisted without any degenerative changes. In the skeletal muscle of mdx mice, necrotic regeneration is observed only up to 1 to 2 months of age, and regenerated myofibers with central nuclei remain for a long time [10, 19, 35]. At present, it is not clear why myofiber necrosis occurs only transiently in mdx mice, but it has been suggested that one reason is that compensatory increases in utrophin, a functional homolog of dystrophin, may replace the function of dystrophin [20]. In the present study, the *Utrn* transcription was higher in the tongue muscle of WT rats than in their masseter muscle. Therefore, one of the possibilities is that some mechanism may be responsible for the increased transcription of *Utrn* in the tongue muscle of the rat, and myofiber necrosis may not occur even under dystrophin deficiency. However, this study only showed differences in transcription levels of utrophin by quantitative RT-PCR, and immunohistochemical analysis to determine the localization, as well as quantitative analysis of protein levels by western blotting, is needed to draw a precise conclusion.

The degree of skeletal muscle regeneration after injury varies depending on the body part; it has been reported that the diameter of myofibers and the degree of fibrosis in masticatory muscles of 100-day-old mdx mice vary according to the muscles [31]. In addition, Yoshioka et al. reported that when injury and subsequent regeneration of skeletal muscle were induced by cardiotoxin injection in WT and mdx mice, there were differences in the rate of regeneration and the degree of increase in muscle weight after regeneration [40]. In particular, the masseter muscle had a longer time to recover its muscle weight after injury than the TA muscle, and no hypertrophy of regenerated myofibers occurred [40]. They suggested that these differences were due to a distinct nature of the satellite cells present in each skeletal muscle. Regarding this, the origin of the satellite cells varies among different muscles [27]. The tongue muscle including satellite cells is thought to be composed of mixed mesodermal origins [23, 27] but detailed convincing lineage tracing experiments of these muscles are not available. On the other hand, the satellite cells of the masseter muscle are derived from the first and second pharyngeal arches of the cranial paraxial mesoderm with contributions from the splanchnic mesoderm [27]. At present, it is unclear how the different origins of these satellite cells ultimately affect the nature of the skeletal muscle from which they arise. However, in the multiple types of myopathies, including DMD, it has been suggested that differences in the origin of satellite cells may contribute to the distinct pathological susceptibility among different muscles [27]. Thus, the phenotypic difference between the masseter and tongue muscles observed in DMD rats may be due to the difference in the nature of the satellite cells. However, we cannot exclude the possibility that the differences in dystrophic changes in each muscle are simply due to differences in the mechanical load on each muscle. In this regard, studies that verify differences in muscle loading in the rat are required.

The tongue muscle has a unique structural feature among skeletal muscles in that it is covered by epithelium, and its development is indirectly influenced by epithelium [14, 18]. During development, the epithelium-derived sonic hedgehog (shh) acts on cranial neural crest cells (CNCC), which constitute the mesenchyme [14]. When the hedgehog signal in CNCC is defective, migration of myoblasts from the occipital somite is perturbed, resulting in dysplasia of the tongue. Although the involvement of the epithelium in the adult tongue has not been reported, it is possible that factors derived from the epithelium act directly or indirectly via mesenchyme on the tongue muscle, which may be protective against degenerative changes of tongue myofibers caused by dystrophin deficiency. This point awaits further investigation.

Macroglossia has been reported to occur not only in human DMD but also in canine X-linked muscular dystrophy (CXMD) [30]. In human DMD, it is known that macroglossia interferes with tongue function [36, 37], but no detailed histological study has been performed. On the other hand, it has been suggested that hypertrophy of myofibers is the cause of macroglossia in CXMD [30]. This is consistent with the results of the present analysis in DMD rats. The mechanisms of skeletal muscle hypertrophy include increased protein synthesis, decreased degradation, and increased number of myonuclei supplied to myofibers by satellite cells [12]. It has been reported that some regenerated myofibers in mdx mice and golden retriever muscular dystrophy (GRMD) dogs show hypertrophy [4, 8, 15, 16, 24, 29], and one of the mechanisms is the vigorous activation of the Akt-mTOR pathway during the period before the occurrence of myofiber necrosis, which results in hypertrophy of myofibers leading to a subsequent reduced severity of pathology [25]. Since the mechanism of myofiber hypertrophy in the tongue has not been analyzed in depth in any of the species reported [28, 30, 37], we attempted to clarify the mechanism of myofiber hypertrophy in the tongue muscle of the rat DMD in the present study. As a result, the signaling molecules involved in the regulation of protein synthesis and degradation were not significantly altered compared to WT rats, at least at the age of 6 months examined in this study. However, this does not necessarily exclude the possibility that the Akt-mTOR pathway is involved in the hypertrophy of tongue myofibers in DMD rats, and it still remains possible that the transient activation of this pathway at a younger age (before 6 months old) had led to the hypertrophy of muscle fibers. On the other hand, although not statistically significant, the number of nuclei per myofiber showed an increasing trend, suggesting that a supply of new myonuclei by satellite cells may have occurred. This is supported by the present result that myogenicity of satellite cells derived from the tongue muscle of DMD rats was relatively preserved in vitro, even at the age of 9 months, unlike those from the masseter muscle.

The limitation of this study is that it is still unclear whether the macroglossia in DMD rats influences tongue function, and it is unknown whether the decreased food intake seen in DMD rats is attributed to macroglossia. Furthermore, it is also possible that the decreased food intake seen in the DMD rats is caused by general fatigue such as respiratory problems or cardiomyopathy. In fact, the DMD rats show extensive fibrosis in their heart [22, 33] and similar distributions and progression of heart involvement to those of patients with DMD [32]. Regarding these points, it is necessary to evaluate the tongue function experimentally and to elucidate the exact cause of decreased food intake in DMD rats in the future.

## Conclusions

We demonstrated that, unlike the masseter and hindlimb muscles, degenerative/necrotic changes of myofibers, decreased satellite cell proliferative activity, and increased p16 and p19 expressions were seldomly observed in the tongue muscle of DMD rats. Furthermore, the tongue muscle of the rat showed higher *Utrn* transcription than the masseter muscle, suggesting that this compensatory *Utrn* transcription might protect the tongue myofibers from degenerative/necrotic changes under the lack of dystrophin. Unraveling the mechanism involved in less advanced dystrophic changes in the tongue muscle in DMD rats would benefit to establish an effective treatment for DMD.

## Supplementary Information

**Additional file 1: Supplementary Fig. 1.** HE-stained sections of the extrinsic tongue muscle of DMD rats. At 3- and 8-month-old, regenerative myofibers with centralized nuclei and basophilic cytoplasm were observed in some genioglossus muscles.

**Additional file 2: Supplementary Fig. 2.** Immunohistochemical analyses of vascular endothelial (CD31) and mesenchymal progenitor (CSPG4) cells. Representative immunohistochemistry of CD31 (red), and CSPG4 (red) and laminin (green) in the tongue muscle of WT and DMD rats at 6-month-old. Nuclei were stained with Hoechst 33258 (blue). Graphed data are quantitative analysis of CD31- and CSPG4-positive areas. The data are expressed as mean+SE (CD31, WT (*n*=5) and DMD (*n*=6); CSPG4, WT (*n*=3) and DMD (*n*=3)).

**Additional file 3: Supplementary Fig. 3.** Western blot of molecules involved downstream of Akt-mTOR pathway in the tongue muscle at 6-month-old. Two bands (doublet) were detected by the anti-4E-BP1 antibody. The upper (p-4E-BP1 (high)) and lower (p-4E-BP1 (low)) bands correspond to highly phosphorylated 4E-BP1 and non- or lower-phosphorylated 4E-BP1, respectively. α-tubulin was used as an internal control. p-Akt (S473), Akt phosphorylated at Ser^473^. p-S6K (T389), S6 kinase phosphorylated at Thr^389^. p-S6 (S240/244), S6 ribosomal protein phosphorylated at Ser^240^ and Ser^244^. n, the number of samples.

**Additional file 4: Supplementary Fig. 4.** Representative immunocytochemistry of satellite cells. The cultured cells were immunostained with anti-Pax7 (A) and anti-MyoD (B) on day 4 of culture. Nuclei were stained with Hoechst 33258. Arrowheads (white) indicate positive cells.

## Abbreviations

ANOVA: Analysis of variance
CNCC: Cranial neural crest cells
CSPG4: Chondroitin sulfate proteoglycan 4
CXMD: Canine X-linked muscular dystrophy
DMD: Duchenne muscular dystrophy
DMD rat: A rat model of DMD
DSHB: Developmental Studies Hybridoma Bank
EDTA: Ethylenediaminetetraacetic acid
eMHC: Embryonic myosin heavy chain
HE: Hematoxylin–eosin
HPRT: Hypoxanthine‐guanine phosphoribosyltransferase
HRP: Horseradish peroxidase
NICHD: National Institute of Child Health and Human Development
NDS: Normal donkey serum
NGS: Normal goat serum
PBS: Phosphate-buffered saline
PFA: Paraformaldehyde
RIPA: Radioimmunoprecipitation assay
SDS-PAGE: Sodium dodecyl sulfate–polyacrylamide gel electrophoresis
Shh: Sonic hedgehog
TA: Tibialis anterior
WT: Wild-type
XY: Male WT
X^Dmd^Y: Male DMD

## References

[1] Ariga M, Nedachi T, Akahori M, Sakamoto H, Ito Y, Hakuno F, Takahashi S (2000). Signalling pathways of insulin-like growth factor-I that are augmented by cAMP in FRTL-5 cells. Biochem J.

[2] Bodine SC, Latres E, Baumhueter S, Lai VK, Nunez L, Clarke BA, Poueymirou WT, Panaro FJ, Na E, Dharmarajan K, Pan ZQ, Valenzuela DM, DeChiara TM, Stitt TN, Yancopoulos GD, Glass DJ (2001). Identification of ubiquitin ligases required for skeletal muscle atrophy. Science.

[3] Botteron S, Verdebout CM, Jeannet PY, Kiliaridis S (2009). Orofacial dysfunction in Duchenne muscular dystrophy. Arch Oral Biol.

[4] Coulton GR, Curtin NA, Morgan JE (1988). The mdx mouse skeletal muscle myopathy: II. Contractile properties. Neuropathol Appl Neurobiol.

[5] da Costa KVT, Ribeiro CMB, de Carvalho FD, Gonçalves LS, de Almeida OP, de Carvalho Silva LT, Bastos YVP, Ferreira SMS (2018). Dysphagia due to macroglossia in a patient with amyloidosis associated with multiple myeloma: a case report. Spec Care Dentist.

[6] Davalli P, Mitic T, Caporali A, Lauriola A, D’Arca D (2016). ROS, cell senescence, and novel molecular mechanisms in aging and age-related diseases. Oxid Med Cell Longev.

[7] Deconinck AE, Rafael JA, Skinner JA, Brown SC, Potter AC, Metzinger L, Watt DJ, Dickson JG, Tinsley JM, Davies KE (1997). Utrophin-dystrophin-deficient mice as a model for Duchenne muscular dystrophy. Cell.

[8] DiMario JX, Uzman A, Strohman RC (1991). Fiber regeneration is not persistent in dystrophic (MDX) mouse skeletal muscle. Dev Biol.

[9] Duan D, Goemans N, Takeda S, Mercuri E, Aartsma-Rus A (2021). Duchenne muscular dystrophy. Nat Rev Dis Primers.

[10] Duddy W, Duguez S, Johnston H, Cohen TV, Phadke A, Gordish-Dressman H, Nagaraju K, Gnocchi V, Low S, Partridge T (2015). Muscular dystrophy in the mdx mouse is a severe myopathy compounded by hypotrophy, hypertrophy and hyperplasia. Skelet Muscle.

[11] Dumont NA, Bentzinger CF, Sincennes MC, Rudnicki MA (2015). Satellite cells and skeletal muscle regeneration. Compr Physiol.

[12] Fukada SI, Ito N (2021). Regulation of muscle hypertrophy: involvement of the Akt-independent pathway and satellite cells in muscle hypertrophy. Exp Cell Res.

[13] Grady RM, Teng H, Nichol MC, Cunningham JC, Wilkinson RS, Sanes JR (1997). Skeletal and cardiac myopathies in mice lacking utrophin and dystrophin: a model for Duchenne muscular dystrophy. Cell.

[14] Jeong J, Mao J, Tenzen T, Kottmann AH, McMahon AP (2004). Hedgehog signaling in the neural crest cells regulates the patterning and growth of facial primordia. Genes Dev.

[15] Kornegay JN, Childers MK, Bogan DJ, Bogan JR, Nghiem P, Wang J, Fan Z, Howard JF, Schatzberg SJ, Dow JL, Grange RW, Styner MA, Hoffman EP, Wagner KR (2012). The paradox of muscle hypertrophy in muscular dystrophy. Phys Med Rehabil Clin N Am.

[16] Kornegay JN, Cundiff DD, Bogan DJ, Bogan JR, Okamura CS (2003). The cranial sartorius muscle undergoes true hypertrophy in dogs with golden retriever muscular dystrophy. Neuromuscul Disord.

[17] Le Révérend BJ, Edelson LR, Loret C (2014). Anatomical, functional, physiological and behavioural aspects of the development of mastication in early childhood. Br J Nutr.

[18] Lin C, Fisher AV, Yin Y, Maruyama T, Veith GM, Dhandha M, Huang GJ, Hsu W, Ma L (2011). The inductive role of Wnt-β-Catenin signaling in the formation of oral apparatus. Dev Biol.

[19] Massopust RT, Lee YI, Pritchard AL, Nguyen VM, McCreedy DA, Thompson WJ (2020). Lifetime analysis of mdx skeletal muscle reveals a progressive pathology that leads to myofiber loss. Sci Rep.

[20] Matsumura K, Ervasti JM, Ohlendieck K, Kahl SD, Campbell KP (1992). Association of dystrophin-related protein with dystrophin-associated proteins in mdx mouse muscle. Nature.

[21] Motohashi N, Asakura A (2014). Muscle satellite cell heterogeneity and self-renewal. Front Cell Dev Biol.

[22] Nakamura K, Fujii W, Tsuboi M, Tanihata J, Teramoto N, Takeuchi S, Naito K, Yamanouchi K, Nishihara M (2014). Generation of muscular dystrophy model rats with a CRISPR/Cas system. Sci Rep.

[23] Parada C, Han D, Chai Y (2012). Molecular and cellular regulatory mechanisms of tongue myogenesis. J Dent Res.

[24] Pastoret C, Sebille A (1995). Mdx mice show progressive weakness and muscle deterioration with age. J Neurol Sci.

[25] Peter AK, Crosbie RH (2006). Hypertrophic response of Duchenne and limb-girdle muscular dystrophies is associated with activation of Akt pathway. Exp Cell Res.

[26] Rafael JA, Tinsley JM, Potter AC, Deconinck AE, Davies KE (1998). Skeletal muscle-specific expression of a utrophin transgene rescues utrophin-dystrophin deficient mice. Nat Genet.

[27] Randolph ME, Pavlath GK (2015). A muscle stem cell for every muscle: variability of satellite cell biology among different muscle groups. Front Aging Neurosci.

[28] Renard D, Humbertclaude V, Labauge P (2010). Macroglossia in adult Duchenne muscular dystrophy. Acta Neurol Belg.

[29] Sacco P, Jones DA, Dick JR (1992). Contractile properties and susceptibility to exercise-induced damage of normal and mdx mouse tibialis anterior muscle. Clin Sci.

[30] Shimatsu Y, Yoshimura M, Yuasa K, Urasawa N, Tomohiro M, Nakura M, Tanigawa M, Nakamura A, Takeda S (2005). Major clinical and histopathological characteristics of canine X-linked muscular dystrophy in Japan. CXMDJ Acta Myol.

[31] Spassov A, Gredes T, Gedrange T, Lucke S, Pavlovic D, Kunert-Keil C (2010). Histological changes in masticatory muscles of mdx mice. Arch Oral Biol.

[32] Sugihara H, Kimura K, Yamanouchi K, Teramoto N, Okano T, Daimon M, Morita H, Takenaka K, Shiga T, Tanihata J, Aoki Y, Inoue-Nagamura T, Yotsuyanagi H, Komuro I (2020). Age-dependent echocardiographic and pathologic findings in a rat model with Duchenne muscular dystrophy generated by CRISPR/Cas9 genome editing. Int Heart J.

[33] Sugihara H, Teramoto N, Nakamura K, Shiga T, Shirakawa T, Matsuo M, Ogasawara M, Nishino I, Matsuwaki T, Nishihara M, Yamanouchi K (2020). Cellular senescence-mediated exacerbation of Duchenne muscular dystrophy. Sci Rep.

[34] Takeuchi S, Nakano S, Nakamura K, Ozoe A, Chien P, Yoshihara H, Hakuno F, Matsuwaki T, Saeki Y, Takahashi S, Yamanouchi K, Nishihara M (2016). Roles of chondroitin sulfate proteoglycan 4 in fibrogenic/adipogenic differentiation in skeletal muscle tissues. Exp Cell Res.

[35] Torres LF, Duchen LW (1987). The mutant mdx: inherited myopathy in the mouse. Morphological studies of nerves, muscles and end-plates. Brain..

[36] van den Engel-Hoek L, de Groot IJ, Sie LT, van Bruggen HW, de Groot SA, Erasmus CE, van Alfen N (2016). Dystrophic changes in masticatory muscles related chewing problems and malocclusions in Duchenne muscular dystrophy. Neuromuscul Disord.

[37] van den Engel-Hoek L, Erasmus CE, Hendriks JC, Geurts AC, Klein WM, Pillen S, Sie LT, de Swart BJ, de Groot IJ (2013). Oral muscles are progressively affected in Duchenne muscular dystrophy: implications for dysphagia treatment. J Neurol.

[38] Waisman A, Norris AM, Elías Costa M, Kopinke D (2021). Automatic and unbiased segmentation and quantification of myofibers in skeletal muscle. Sci Rep.

[39] Yamanouchi K, Nakamura K, Takeuchi S, Hosoyama T, Matsuwaki T, Nishihara M (2021). Suppression of MyoD induces spontaneous adipogenesis in skeletal muscle progenitor cell culture. Anim Sci J.

[40] Yoshioka K, Kitajima Y, Seko D, Tsuchiya Y, Ono Y (2021). The body region specificity in murine models of muscle regeneration and atrophy. Acta Physiol (Oxf).

