# Supplementary figures and images for: Macroglossia and less advanced dystrophic change in the tongue muscle of the Duchenne muscular dystrophy rat
Source: Skelet Muscle. 2022 Oct 19;12:24. doi: 10.1186/s13395-022-00307-7 (PMC9580129; doi:10.1186/s13395-022-00307-7)

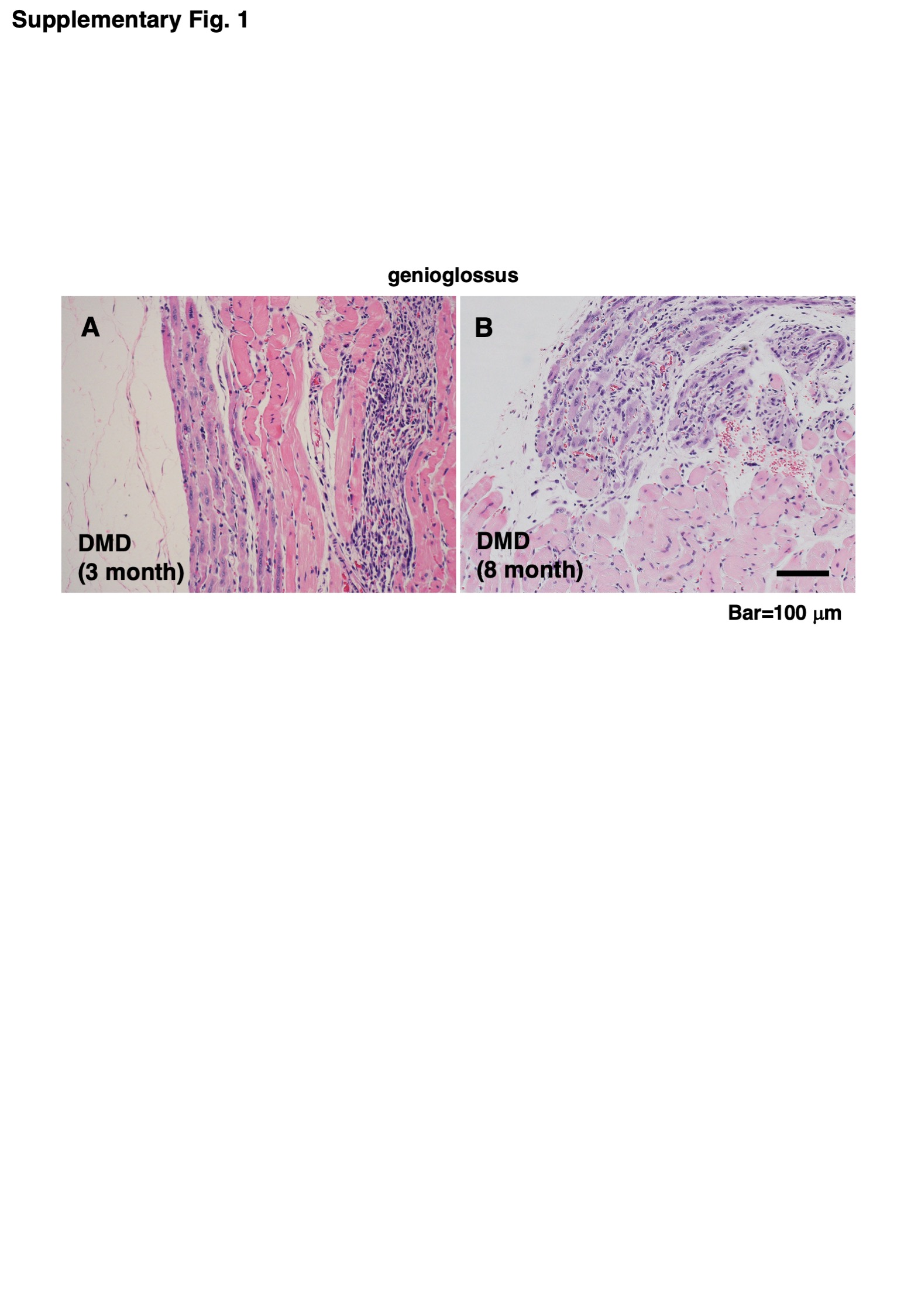

Supplement: Supplementary file 1 — Additional file 1: Supplementary Fig. 1. HE-stained sections of the extrinsic tongue muscle of DMD rats. At 3- and 8-month-old, regenerative myofibers with centralized nuclei and basophilic cytoplasm were observed in some genioglossus muscles. [file 13395_2022_307_MOESM1_ESM.jpg]

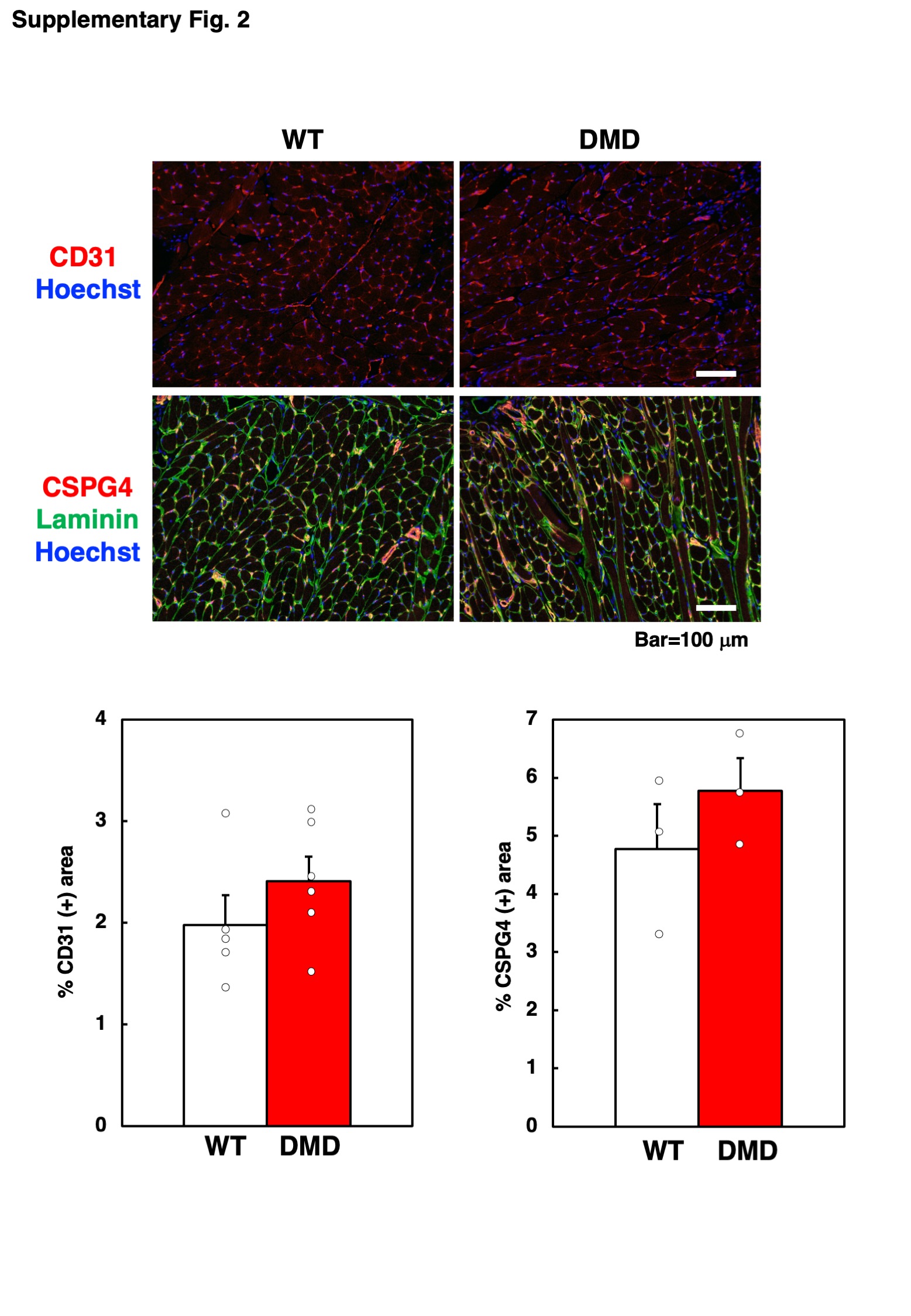

Supplement: Supplementary file 2 — Additional file 2: Supplementary Fig. 2. Immunohistochemical analyses of vascular endothelial (CD31) and mesenchymal progenitor (CSPG4) cells. Representative immunohistochemistry of CD31 (red), and CSPG4 (red) and laminin (green) in the tongue muscle of WT and DMD rats at 6-month-old. Nuclei were stained with Hoechst 33258 (blue). Graphed data are quantitative analysis of CD31- and CSPG4-positive areas. The data are expressed as mean+SE (CD31, WT (n=5) and DMD (n=6); CSPG4, WT (n=3) and DMD (n=3)). [file 13395_2022_307_MOESM2_ESM.jpg]

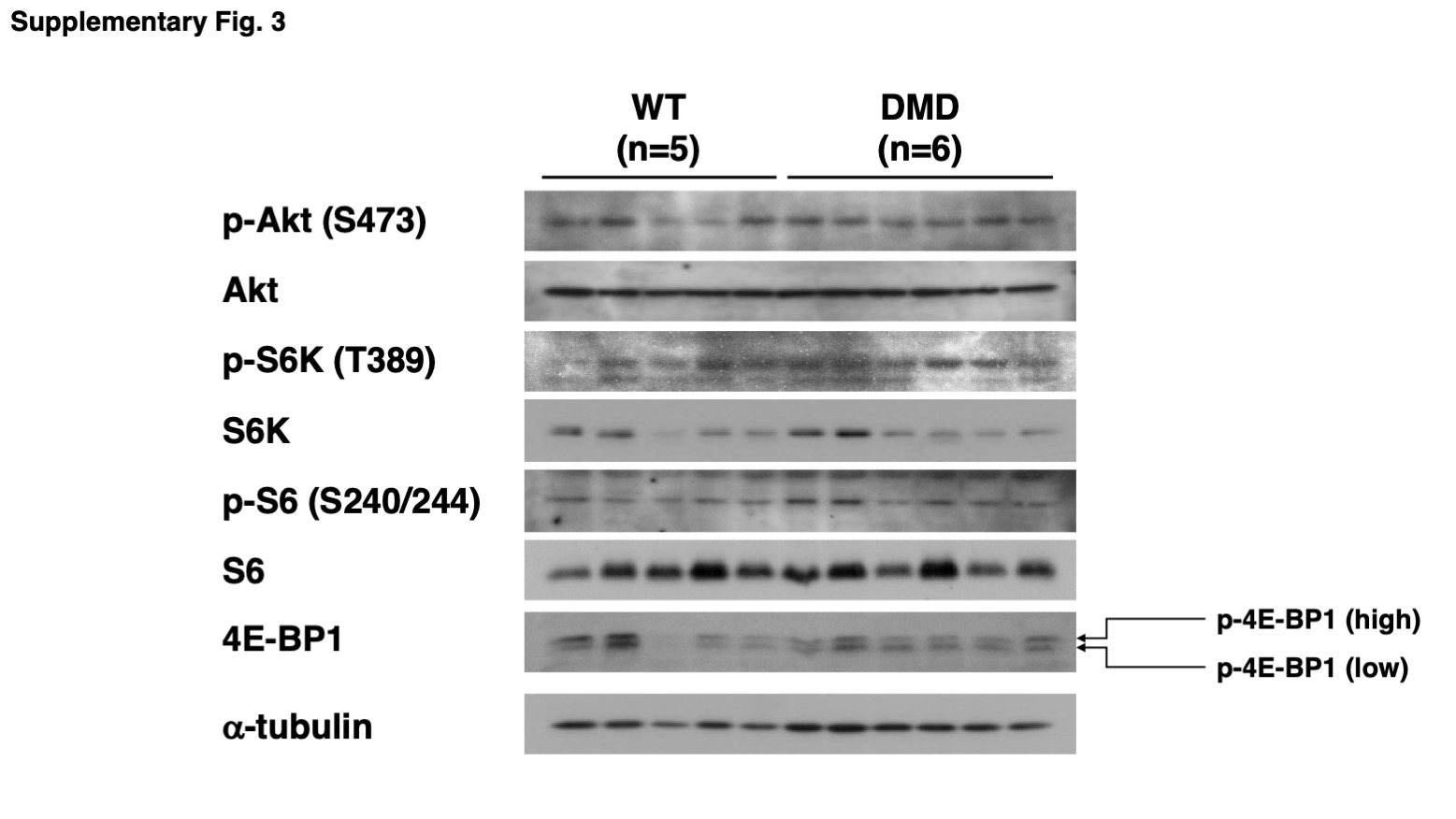

Supplement: Supplementary file 3 — Additional file 3: Supplementary Fig. 3. Western blot of molecules involved downstream of Akt-mTOR pathway in the tongue muscle at 6-month-old. Two bands (doublet) were detected by the anti-4E-BP1 antibody. The upper (p-4E-BP1 (high)) and lower (p-4E-BP1 (low)) bands correspond to highly phosphorylated 4E-BP1 and non- or lower-phosphorylated 4E-BP1, respectively. α-tubulin was used as an internal control. p-Akt (S473), Akt phosphorylated at Ser473. p-S6K (T389), S6 kinase phosphorylated at Thr389. p-S6 (S240/244), S6 ribosomal protein phosphorylated at Ser240 and Ser244. n, the number of samples. [file 13395_2022_307_MOESM3_ESM.jpg]

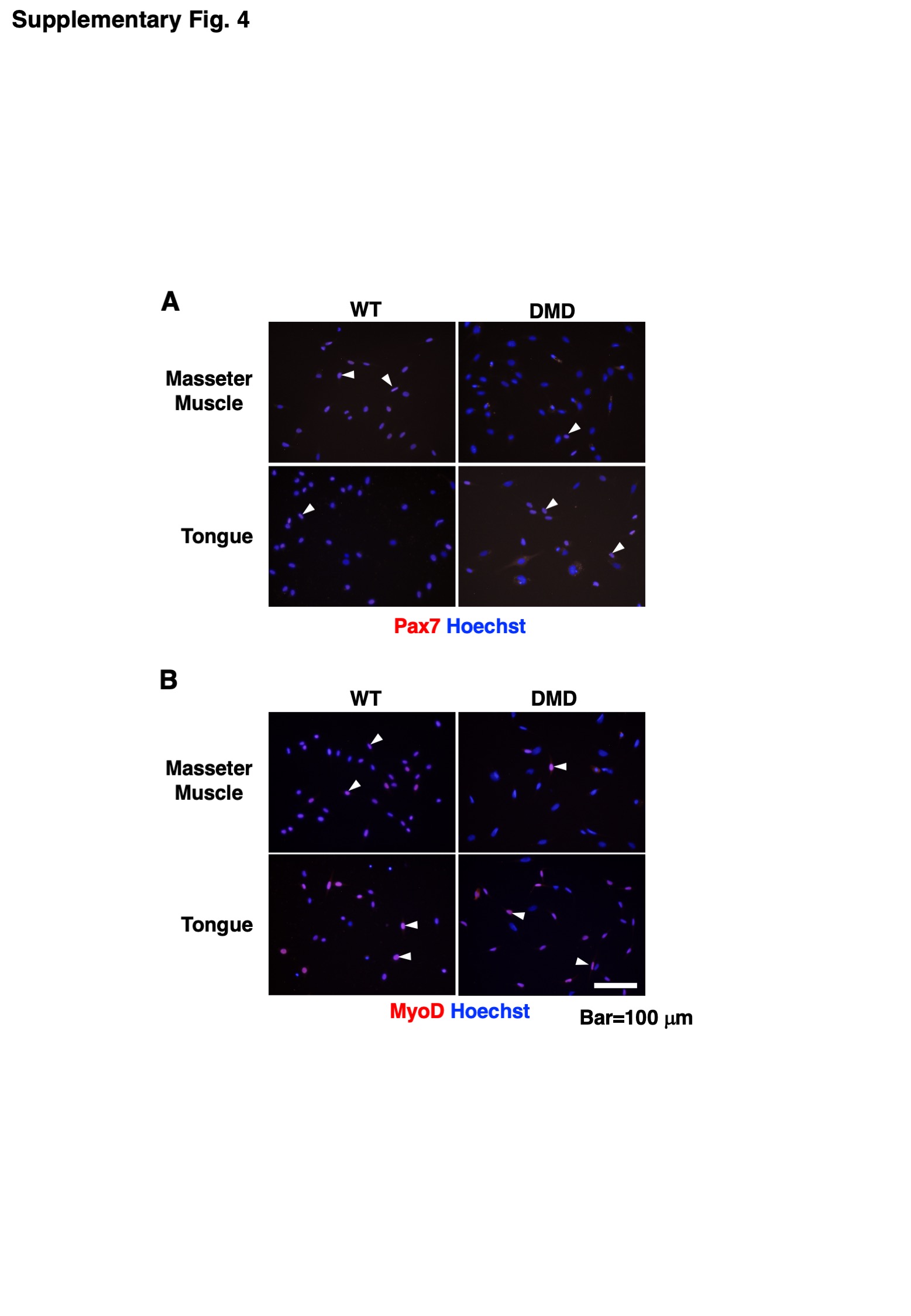

Supplement: Supplementary file 4 — Additional file 4: Supplementary Fig. 4. Representative immunocytochemistry of satellite cells. The cultured cells were immunostained with anti-Pax7 (A) and anti-MyoD (B) on day 4 of culture. Nuclei were stained with Hoechst 33258. Arrowheads (white) indicate positive cells. [file 13395_2022_307_MOESM4_ESM.jpg]
